# Metabolic Pathways Involved in Regulatory T Cell Functionality

**DOI:** 10.3389/fimmu.2019.02839

**Published:** 2019-12-03

**Authors:** Rosalie W. M. Kempkes, Irma Joosten, Hans J. P. M. Koenen, Xuehui He

**Affiliations:** Laboratory of Medical Immunology, Department of Laboratory Medicine, Radboud University Medical Center, Nijmegen, Netherlands

**Keywords:** metabolism, human Treg cells, FOXP3, proliferation, migration, suppressive function, tolerance-inducing therapies

## Abstract

Regulatory T cells (Treg) are well-known for their immune regulatory potential and are essential for maintaining immune homeostasis. The rationale of Treg-based immunotherapy for treating autoimmunity and transplant rejection is to tip the immune balance of effector T cell-mediated immune activation and Treg-mediated immune inhibition in favor of Treg cells, either through endogenous Treg expansion strategies or adoptive transfer of *ex vivo* expanded Treg. Compelling evidence indicates that Treg show properties of phenotypic heterogeneity and instability, which has caused considerable debate in the field regarding their correct use. Consequently, for further optimization of Treg-based immunotherapy, it is vital to further our understanding of Treg proliferative, migratory, and suppressive behavior. It is increasingly appreciated that the functional profile of immune cells is highly dependent on their metabolic state. Although the metabolic profiles of effector T cells are progressively understood, little is known on Treg in this respect. The objective of this review is to outline the current knowledge of human Treg metabolic profiles associated with the regulation of Treg functionality. As such information on human Treg is still limited, where information was lacking, we included insightful findings from mouse studies. To assess the available evidence on metabolic pathways involved in Treg functionality, PubMed, and Embase were searched for articles in English indexed before April 28th, 2019 using “regulatory T lymphocyte,” “cell metabolism,” “cell proliferation,” “migration,” “suppressor function,” and related search terms. Removal of duplicates and search of the references was performed manually. We discerned that while glycolysis fuels the biosynthetic and bioenergetic needs necessary for proliferation and migration of human Treg, suppressive capacity is mainly maintained by oxidative metabolism. Based on the knowledge of metabolic differences between Treg and non-Treg cells, we additionally discuss and propose ways of how human Treg metabolism could be exploited for the betterment of tolerance-inducing therapies.

## Introduction

To safeguard ourselves, our immune system is equipped with a series of defense systems to recognize and respond to *non-self*-structures. Although essential for fighting off infections and preventing cancers from arising, destructive immune responses pose a considerable challenge in autoinflammation and transplantation. Currently available immunosuppressants help to control destructive immune responses. However, management of side-effects of lifelong immunosuppression, including cancer development and reduced survival, remain major problems (1). For this reason, an increasing amount of interest is directed toward the natural specific regulatory mechanism of the immune system. A better understanding of these mechanisms holds the key to the development of novel immunomodulatory therapies (2).

**Figure 1:**
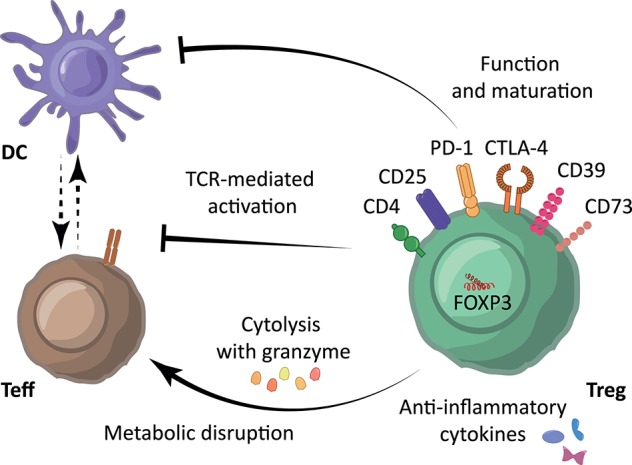
Immunosuppressive mechanisms underlying Treg-mediated immune suppression. Treg are characterized by expression of the cell surface markers CD4^+^, CD25^high^ and CD127^low/−^, and transcription factor FOXP3. Treg modulate the immune system using their suppressive molecules PD-1, CTLA-4, CD39, and various surface receptors through inhibition of dendritic cell (DC) function and maturation, through the secretion of anti-inflammatory cytokines such as IL-10, TGF-β and IL-35, and/or through direct inhibition of Teff via induction of cytolysis using granzyme and metabolic disruption. Moreover, Treg can reduce Teff activation by limiting TCR-ligand binding.

One approach the immune system employs to induce *self*-tolerance is via regulatory T cells (Treg). Treg modulate the immune system both specifically and aspecifically via inhibition of dendritic cell function and maturation, secretion of anti-inflammatory cytokines, and suppression of induction and proliferation of antigen-specific effector T cells (Teff) (3), depicted in Figure 1. As reviewed previously (4), human Treg are characterized by expression of the transcription factor Forkhead box p3 (FOXP3) and the combination of cell surface markers CD4^+^, CD25^+^, and CD127^low/−^. FOXP3 is the most reliable cell marker for Treg, although it is also transiently expressed by activated effector T cells. Nevertheless, FOXP3 expression is essential for the maturation and function of Treg. Suppressive capacity of Treg is commonly associated with the amount of FOXP3 expression. Also strength, binding and expression of the T cell receptor (TCR), capacity to produce anti-inflammatory cytokines like interleukin-10 (IL-10) and IL-35, and expression of suppressive cell membrane molecules like cytotoxic T-lymphocyte-associated protein 4 (CTLA-4), programmed cell death protein 1 (PD-1), CD39, and CD73 are fundamental in this (3), as depicted in Figure 1. Of note, expression levels from some of these molecules have been linked to metabolism. Following Treg activation, mTOR signaling is upregulated which induces lipid synthesis, mevalonate metabolism and mitochondrial function (5). CDTLA-4 and PD-1 have both been described to block glycolysis whereby PD-1 signaling also promotes lipolysis and fatty acid oxidation (6). CD39, which converts adenosine triphosphate (ATP) and adenosine diphosphate (ADP) into adenosine monophosphate (AMP), and CD73, which subsequently converts AMP to adenosine, abrogates ATP-effects such as P2 receptor-mediated cell toxicity and ATP-driven maturation of dendritic cells.

Human Treg are currently intensively studied for the induction of immunotolerance both in transplantation and autoimmunity (7). Although these studies have substantially advanced our knowledge on Treg, important issues on the optimization of these therapies regarding Treg expansion, homing and stability of immunoregulatory function remain to be resolved. Here, we asked ourselves whether employing knowledge on cell metabolism can be of benefit for the advancement of Treg therapy.

**Figure 2:**
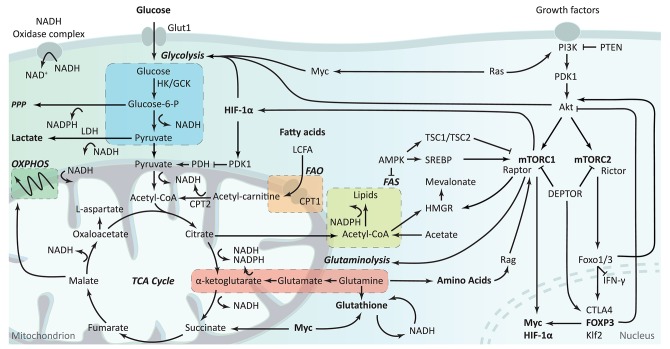
Overview of cellular metabolism in T cells. The first step of glycolysis is the conversion of glucose into glucose-6-phosphate by hexokinases (HK) or glucokinase (GCK). During glycolysis, glucose can be shuttled away toward the pentose phosphate pathway (PPP), or stay in glycolysis resulting in its conversion into pyruvate. Pyruvate can either be converted to lactate by lactate dehydrogenase (LDH), using the nicotinamide adenine dinucleotide (NADH) produced during glycolysis, or it can be converted into acetyl-CoA and enter the tricarboxylic acid (TCA) cycle. The TCA cycle can also be fueled by acetyl-CoA produced during FAO and α-ketoglutarate following glutaminolysis. Subsequently, the TCA cycle fuels OXPHOS and FAS. NADH and reduced nicotinamide adenine dinucleotide phosphate (NADPH) are produced during the conversion of various metabolites and play an important role in proton homeostasis and ROS production via the NADP^+^ complex and glutathione metabolism. Although all these metabolic pathways are interlinked, cells can regulate their isolated activity and compensate for alterations in the different pathways. The PI3K-Akt-mTOR pathway plays a role in metabolic regulation through various routes. Glycolysis is stimulated via Akt and HIF-1α while fatty acid synthesis and amino acid synthesis are impacted by and impact mTORC1. The PI3K-Akt pathway also modulates FOXP3 expression, which itself impact metabolism via Myc and Akt signaling. OXPHOS, oxidative phosphorylation; FAO, fatty acid oxidation; FAS, fatty acid synthesis.

Cell metabolism was shown to be a fundamental determinant of immunological cell fate and function (8). Most resting immune cells are relatively metabolic inactive. However, during activation, immune cells increase their metabolic requirements and switch their metabolic programs to accommodate the increased demand for energy and biosynthesis (8). Although glycolysis is a rapid method for energy production and it supports other metabolic pathways by producing nicotinamide adenine dinucleotide (NADH), it is a relatively inefficient energy source compared to the mitochondrial oxidative metabolism of oxidative phosphorylation (OXPHOS) (Figure 2). In glycolysis, glucose is converted in the cytoplasm into glucose-6-phosphate by hexokinase (HK) or glucokinase and subsequently converted into pyruvate or shuttled into the pentose phosphate pathway (PPP). During the PPP, nicotinamide adenine dinucleotide phosphate (NADPH), a precursor for the synthesis of nucleotides and amino acids, is produced. Pyruvate resulting from glycolysis can either be converted into lactate by lactate dehydrogenase (LDH) or converted into acetyl-CoA to enter the tricarboxylic acid (TCA) cycle. The TCA cycle, also known as the citric acid cycle or the Krebs cycle, is the primary metabolic pathway of quiescent or non-proliferating cells and a highly efficient source of energy. Besides acetyl-CoA from pyruvate, the TCA cycle is also fueled by acetyl-CoA produced during fatty acid oxidation, α-ketoglutarate following glutaminolysis, and by the acids citrate and succinate. These can subsequently proceed into OXPHOS, which is a less rapid but more energy-efficient pathway. Acetyl-CoA from glycolysis and fatty acid oxidation (FAO) can also support the mevalonate pathway. OXPHOS follows fatty acid oxidation and the TCA cycle can be followed by fatty acid synthesis. Both glycolysis and fatty acid oxidation support the mevalonate pathway via acetyl-CoA.

Generally, glycolysis tends to support the function of pro-inflammatory cells, such as Teff and M1 macrophages, while OXPHOS and FAO tend to be used by anti-inflammatory cells, such as M2 macrophages, memory CD8^+^ Teff and Treg. Resting T cells mostly utilize a catabolic oxidative metabolism of glucose, lipids, and amino acids, whereas, upon activation T cells upregulate their glucose and amino acid transporters and increase their glycolytic flux to fuel the enhanced glycolysis process, thus supporting their proliferation and inflammatory functions. While the metabolic requirements for conventional T cells are increasingly understood, those important for Treg have been less well-defined. Mostly, Treg are assumed to rely more on the TCA cycle and OXPHOS than on glycolysis, leaving the complex interplay between metabolism and functional state underexposed. Thus, a better insight into Treg metabolism and its relevance for tolerance-inducing therapies could benefit further improvement of tolerance-inducing therapies. The characterized metabolic pathways involved in Teff and Treg are summarized in Table 1. In this review, we will detail the contribution of these metabolic pathways to Treg functionalities with the focus on cell proliferation, migration and suppressive capacity. Furthermore, we will discuss exploiting Treg metabolism for tolerance-inducing therapies. Although the main focus of this review is on human Treg, we also included insightful findings from mouse studies in cases where human information was lacking.

**Table 1:** Characterized metabolic pathways involved in human effector and regulatory T cells.

	**Effector T cells (Teff)**	**Regulatory T cells (Treg)**
Metabolic pathways in quiescent cells	Catabolic oxidative metabolism of glucose, lipids, and amino acids	Fatty acids oxidation (FAO) OXPHOS
Energy sources upon activation	Aerobic glycolysis Mitochondrial respiration	Mitochondrial oxidation of lipids, and pyruvate *in vitro* and *in vivo* Highly glycolytic *in vivo*
Glucose transporter	High GLUT1	Low GLUT1
Glutaminolysis	Critical for Th1/Th17 differentiation	Inhibits iTreg generation
Amino acids metabolite	Crucial for Teff proliferation and cell survival	Promote iTreg differentiation Crucial for Treg function
Mitochondrial mass and ROS	Low	High
Fatty acids	Increase glycolytic flux Promotes Th1/Th17 differentiation	SCFAs promote Treg differentiation and function
PPARγ	Inhibits Th17 differentiation	Maintenance and accumulation of Treg in adipose tissue
PI3k/Akt/mTOR signals	High (upon activation)	Low (upon activation)
Basal level of mTORC1 activation	Low	High
Signals via AMPK	Low Pro-survival function of AMPK	High Promotes Treg generation
Signals via Myc and HIF-1α	High (upon activation) Promote Th17 differentiation	Low (upon activation) Impairs iTreg generation and Treg lineage stability

*AMPK, AMP-activated protein kinase; mTORC1, mTOR complex 1; PPAR, peroxisome proliferator-activated receptors; SCFA, short-chain fatty acids*.

## Methods

To assess available evidence on metabolic pathways involved in human Treg functionality, PubMed, and Embase were searched for articles indexed in English before April 28th, 2019 for “regulatory T lymphocyte,” “cell metabolism,” “cell proliferation,” “migration,” “suppressor function,” and related search items (Supplementary Table S1). Where studies on human Treg metabolic profiles were lacking, we included insightful findings from mouse studies. Removal of duplicates and search of references was performed manually.

## Metabolic Pathways Linked to Treg Behavior and Function

### Proliferative Behavior and Development of Treg

To manage the accompanying increased demand for energy and biosynthesis during cell growth, the engagement of specific metabolic pathways is essential. Proliferative cells have typically high glycolytic rates and employ the glycolysis-diverting pathways PPP and serine biosynthesis pathway for energy while utilizing glutamine as fuel for the synthesis of biomass (Figure 2). In cancer cells, this switch is referred to as the Warburg effect (9). A similar phenotype has been described for T cells (10).

Glucose is the major source for generating ATP. Glucose can be broken down via either the glycolysis process, i.e., in the cytoplasm converting glucose to pyruvate which is further diverted to lactate by LDH, or the TCA cycle when pyruvate dehydrogenase (PDH) converted pyruvate to acetyl-CoA. Upon activation, T cells preferentially use glycolysis for producing ATP to meet their rapid expansion.

In contrast to Teff cells, which mainly use glycolysis following stimulation, memory T cells, as well as Treg, have a quite different metabolic state, since they largely rely on the FAO for cell persistence and function. Pro-growth signaling pathways, including phosphatidylinositol 3-kinase (PI3K), mitogen-activated protein kinase (MAPK), as well as mammalian target of rapamycin (mTOR) promote glycolysis in Treg (11). Glucose uptake in Treg is mostly mediated by differential expression of the glucose transporter GLUT1. Interestingly, Glut1 expression is higher on activated murine induced Treg (iTreg) than proliferated tTreg, and Glut1 enhances Treg proliferation but inhibits their suppressive function. Notably, GLUT1 expression is reduced by the Treg transcription factor FOXP3. tTreg from mice with transgenic T cell-specific expression of Glut1 showed impaired lineage stability as they had decreased expression of CD25/EZH2 and secreted IFNγ (11). Inhibition of mTOR in Treg greatly diminishes glucose uptake, but it helps to maintain a stable Treg phenotype and high suppressive capacity (12). Depletion of glucose is detrimental for Treg proliferation since Treg mitochondrial oxidize lipid as well as glycolysis derived pyruvate at high speed (13). Of interest, human carriers of a loss-of-function glucokinase (GCK) regulatory protein gene that leads to an enhanced GCK activity, have reduced circulating Treg numbers (14), indicating that either Treg proliferation is impaired or the Treg have migrated to tissue sites. Moreover, evidence showed that inhibition of glycolysis could steer T cells toward Treg differentiation (15), and this happens by increasing FOXP3 expression via the inhibition of mTOR-mediated induction of the transcription factor hypoxia-inducible factor-1α (HIF-1α) (15, 16).

Actively proliferating T cells change from a catabolic metabolism to an anabolic metabolism, in which the fatty acids and amino acids are shunted away from the TCA cycle into membrane and protein synthesis, respectively (Figure 2). HIF-1α plays an important role in the decision to commit pyruvate to lactate for glycolysis or to acetyl-CoA for entering the TCA cycle. HIF-1α induces the expression of glycolytic genes and increases LDH enzyme activity, thus enhancing the conversion of pyruvate to lactate and leading to a glycolysis-shift. It also actively represses mitochondrial function via the induction of pyruvate dehydrogenase kinase 1 (PDK1) (17, 18). PDK1 inhibits PDH and thereby reduces the mitochondrial oxidization of glycolysis-derived pyruvate via the transformation of pyruvate to acetyl-CoA, which acts as the intramitochondrial starting point of OXOPHOS (Figure 2). Inhibition of PDK1 increases Treg numbers in mice (19), which is independent of PDK itself, but through the production of reactive oxygen species (ROS), potentially due to the increased capacity of Treg to scavenge ROS away compared to other T cell subsets (13). Both nTreg and iTreg have higher mitochondrial mass and higher ROS production which are correlated with increased FOXP3 expression (13). ROS is produced following the TCA cycle during subsequent OXPHOS. Although ROS plays important roles in cell signaling and homeostasis, inhibition of OXPHOS does not affect Treg differentiation (20).

Although Treg mainly use oxidative metabolism for growing, Treg proliferation is dependent on an oscillatory switch of glycolysis (21). In mice, both glycolysis and OXOPHOS are involved in the generation of iTreg as well as the growth of Teff cells (13). Freshly isolated human Treg show a high metabolic state including increased mTOR pathway, high amount of phospho-STAT5, and hyporesponsiveness *in vitro*. Interestingly, transient inhibition of mTOR, before TCR stimulation, promotes TCR-induced Treg proliferation, while later (60–72 h after stimulation) actively proliferating Treg cells displayed high mTOR activity (21). It seems that Treg proliferation is closely associated with the dynamic changes in mTOR activity which is influenced by the composition of nutrients within the extracellular milieu.

Treg exhibit increased FAO and enhanced expression of genes involved in FAO during proliferation (19, 22). FAO is a multistep process in which the long-chain fatty acids are first conjugated to carnitine via the rate-limiting enzyme carnitine palmitoyltransferase 1 (CPT1). These are subsequently shuttled to the mitochondrion and converted to acyl-CoA by carnitine palmitoyltransferase 2 (CTP2). This acyl-CoA is further degenerated by β-oxidation to produce acetyl-CoA, which then enters the TCA cycle. CPT1 inhibitor etomoxir inhibits Treg, but not Teff differentiation and proliferation, demonstrating a selective dependency of Treg on FAO. Both murine tTreg and iTreg show higher mitochondrial mass and increased ROS production as compared to Teff or non-T cells (13). Upstream, activated AMPK release the inhibition of CPT1 thus allowing the transport of long-chain fatty acids to the mitochondria for subsequent FAO and ATP generation. Of interest, Treg show high levels of activated AMPK and treatment with metformin, an indirect activator of AMPK, can decreases total T cell numbers, while increasing the percentage and number of Treg (23). Leptin, a cytokine-like hormone stimulating FAO and glucose uptake, constrains the proliferation of Treg through AMPK and mTOR activation (24). Transient inhibition of the leptin-mTOR pathway promotes TCR-induced proliferation in Treg, while an intact mTOR pathway is needed to sustain Treg proliferation (21). This seeming contradiction might be explained by a need for a lower metabolic rate to enter the cell cycle and start proliferation in Treg. In fact, it is shown that *in vitro* proliferation of human Treg requires both glycolysis and FAO (25).

The dependence of Treg on FAO is more evident in tissue-resident Treg, such as visceral adipose tissue (VAT) Treg and intestinal mucosa Treg. VAT Treg are specifically recruited to adipose tissue to suppress the local inflammatory process (26). VAT Treg uniquely express PPARγ (peroxisome proliferator-activated receptors γ), which is crucial in peroxisomal-mediated β-oxidation of FAO, and show a high expression of CD36, a receptor that facilitates the import of fatty acids. Like other Treg, VAT Treg also express leptin-receptors. Leptin binding to its receptor would lead to high activation of mTOR, which affects Treg proliferation. In mice, it has been shown that adipose tissue of obese mice contains high levels of leptin, associated with decreased numbers of Treg, as opposed to lean mice (27). Another example of the effect of anatomical location on Treg metabolism comes from mucosal Treg. The intestinal environment is known to be rich in short-chain fatty acids, such as propionate and butyrate that are generated from the fermentation of dietary fiber. The short-chain fatty acids have been described to influence Treg numbers *in vivo*. Butyrate enhances histone H3 acetylation in the promoter region of FOXP3 and thereby boosts extrathymic Treg formation (28). Additionally, butyrate appears to mediate Treg differentiation by engagement with gut-epithelial cells that subsequently produce IL-10 and a range of metabolites, including retinoic acid, which leads to the production of inducible Treg (29). Propionate, which is also capable of increasing histone acetylation, also promotes Treg generation, while acetate cannot increase histone acetylation and promote Treg generation (30).

Amino acids are used as substrates in various metabolic pathways. Most noteworthy, glutamic acid derivates such as glutamine and glutamate fuel the TCA cycle and scavenge ROS. Correspondingly, the availability and metabolism of amino acids play a decisive role in iTreg generation. Although Treg have lager reserve pools of reduced and oxidized glutathione to cope with higher levels of ROS in Treg, deprivation of glutamine steers Treg generation even in conditions favoring other T cell subsets, whereas supplementation with glutamine supports differentiation toward Th1 (31). Similarly, depletion of arginine and tryptophan *in vitro* cell cultures stimulates Treg generation (32). Interestingly, the byproduct of tryptophan catabolism seems to benefit the iTreg generation since the administration of tryptophan enhances the number of Treg (33). Moreover, Treg are known to highly express amino acids catabolizing enzymes arginase 1 (ARG1) that is responsible for the depletion of extracellular L-arginine thus limiting T cell proliferation, suggesting that Treg could sense the concentration of certain amino acids and/or their byproducts in the local milieu and thereby adjusting the suppressive function properly.

**Figure 3:**
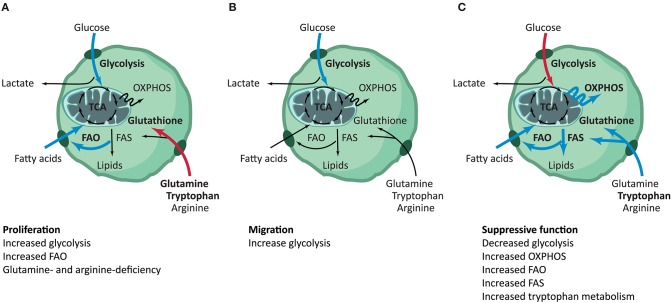
Summary of metabolic pathways involved in distinct Treg functionalities. Treg have distinct metabolic phenotypes throughout their different phases, although many pathways remain to be elucidated. **(A)** During proliferation, Treg have increased glycolysis and FAO. Deficiency of glutamine and tryptophan steers T cells toward Treg differentiation (10–31). **(B)** To support the increased need for energy during migration, Treg increase their glycolytic flux. Metabolic shifts in other pathways have not been described for Treg migration (15, 16, 33–39). **(C)** Treg show decreased glycolysis and increased OXPHOS, FAO, FAS and tryptophan metabolism during their phase of suppressive function. No relevance for the TCA cycle has been reported (6, 10, 14–17, 21, 25, 26, 29, 31, 40–53). The blue and red arrows are indicative for increased or decreased activity of the specific pathway in the functional phenotype of Treg, respectively. FAO, fatty acid oxidation; FAS, fatty acid synthesis; TCA, tricarboxylic acid; OXPHOS, oxidative phosphorylation.

In summary, overall evidence suggests that, compared to other T cell subsets, Treg appear to demonstrate a selective dependency on FAO during proliferation, while being less dependent on glycolysis (Figure 3A). As compared to Teff, in general, Treg show a higher level of lipid oxidization as well as the mitochondrial oxidization of glucose. Glucose is required for Treg cell growth and is a key requirement at the early phase of iTreg generation. Activated iTreg expressed highest GLUT1 transporter, although it is still quite low as compared to non-Treg cells, than proliferated tTreg. Blocking of glycolysis seems to promote the generation of iTreg through mTOR-mediated regulation of HIF-1α, which in turn prevents the mitochondrial oxidation of glucose thus leading to a glycolysis shift. Like non-Treg cells, Treg adapt to their environmental nutrient and oxygen status via the opposing actions of mTOR, AMPK, and HIF-1α. Conditions that decrease mTOR activation permit FOXP3 expression, which in turn re-programs T cells to enhance the expression of genes involved in FAO. The oscillatory changes of leptin-mTOR pathways (early downregulation of mTOR activity followed by a full activation of mTOR pathway during Treg expansion) seem to set the threshold for Treg proliferation. FAO is especially crucial to keep the optimal number of tissue-resident Treg, and the metabolism of amino acids is crucial for the generation of iTreg.

### Migratory Behavior of Treg

Proper orchestration of immune responses and suppression thereof requires appropriate control of Treg migration within both lymphoid and non-lymphoid organs. As reviewed by Chow et al., mechanisms of Treg migration are diverse and differ according to their developmental stage, role and tissue target (34). To be able to regulate immune responses when and where needed, tight regulation of expression of adhesion molecules and chemokines receptors according to the developmental stage and the microenvironment of the Treg is essential. Although migration is likely the most energy-consuming cellular activity, the metabolic demands for Treg locomotion are poorly investigated.

Like most migratory cells, Treg engage in glycolysis to meet their bioenergetic needs for migration. Treg motility can be inhibited by depletion of glucose from the culture medium, inhibition of glucose uptake or inhibition of glycolysis. To support their increased glucose consumption, Treg upregulate their insulin receptor (INSR) (35). Kishore et al. have demonstrated that Treg migration requires GCK activation for the conversion of glucose in the activation of glycolysis (14) (also see Figure 2). GCK promotes migration following pro-migratory and pro-glycolytic stimuli via PI3K-mTORC2 by cytoskeletal rearrangements and by associating with the cytoskeleton component actin. GCK contribution to human Treg migration was observed in human carriers of a loss-of-function polymorphism in the GCKR gene (C to T, P446L), which lead to an increased GCK activity. 446L-GCKR Treg displayed increased chemokine-induced motility compared to WT-GCKR Treg, although the suppressive ability and phenotype did not significantly differ from WT-GCKR Treg (14).

Treg migration is also regulated by glycolytic feedback control through PI3K-Akt pathways (36). Recent investigations of Finlay et al. have established that activation of Akt downregulates the expression of leukocyte adhesion molecule L-selectin (CD62L), chemokine receptors C-C chemokine receptor 7 (CCR7), and Sphingosine-1-phosphate receptor 1 (S1PR1) by control of Foxo1 and Foxo3 (37). Therefore, Akt activation could result in failure of leukocyte-homing to secondary lymphoid organs and stimulate migration to peripheral tissues in humans (38). Exposure of Treg to the mTOR inhibitor rapamycin suppresses upregulation of both α4β7 and CCR9, suggesting that the mechanism is mTOR-dependent. Strikingly, rapamycin-insensitive companion of mTOR (RICTOR) or (mTORC2)-deficient Treg have unaltered ability to express CCR9, while RAPTOR(mTORC1)-deficient Treg are unable to upregulate CCR9, suggesting the selective participation of mTORC1 in the regulation of Treg motility (39). Loss of phosphatase and tensin homolog (PTEN) also impacts migration by lowering CD62L and CCR7 expression (54). This is suggested to be mediated by master kinase phosphoinositide-dependent kinase-1 (PDK1) signaling, which has an important role in the signaling pathways activated by several growth factors and hormones, including insulin signaling. To retain glycolytic flux, pyruvate is often converted into lactate (Figure 2). Extracellular sodium-lactate and lactic acid have been described to entrap CD4^+^ and CD8^+^ T cells at sites of infection by repressing their migratory capacity (55). Lactate mediated inhibition of motility both *in vitro* and *in vivo* appears to be caused by interference with glycolysis. This selective control of motility is mediated by the specific monocarboxylate transporter Slc5a12, which amongst others transports lactate and pyruvate across the cell membrane (55). Glycolysis is otherwise activated upon engagement of the chemokine receptor CXCR3 with its ligand CXCL10, stimulating lymphocyte tissue infiltration.

There is limited to no sufficient empirical evidence for metabolic control of Treg migration through the TCA cycle and OXPHOS. Neither engagement of a central mediator of T cell migration, lymphocyte function-association 1 (LFA-1), nor inhibition of fatty acid oxidation impacts Treg migration (14). However, the lipid-activated S1PR1 was reported to induce selective activation of the Akt-mTOR kinase pathway in Treg migration from lymphoid organs to blood (40). Interestingly, although Treg depend similarly on S1PR1 as Teff, S1PR1 drives Treg accumulation in tumors, but not CD8^+^ T cells (41). It is known that the tumor microenvironment is enriched with indoleamine-pyrrole 2,3-dioxygenase (IDO), which metabolizes tryptophan to kynurenine, an endogenous ligand for the aryl hydrocarbon receptor (AHR). IDO could reduce local tryptophan availability in the proximity of Treg thus contributing to their motility (42).

Summarizing, glycolysis is essential to support the bioenergetic needs of Treg migration, like in most migratory cells. mTORC2 plays a non-redundant role in the regulation of Treg motility, probably through regulating the PI3K-Akt pathway, as well as GCK kinase activity to mediate cytoskeleton reorganization (Figure 3B).

### Suppressive Function and Stability of Treg

To exert their suppressive function, Treg stability, and therefore stable FOXP3 expression is imperative. The stability of Treg is currently the topic of many studies in the Treg field (4). Importantly, besides the loss of suppressive function, Treg can differentiate into proinflammatory cytokine-producing cells (also named exTreg) under specific microenvironmental cues, and induce detrimental immune responses, posing a threat for adoptive Treg therapies. Several transcriptional programs are involved for activated Treg further differentiation into a suppressive effect state. For instance, the transcription factor interferon regulatory factor 4 (IRF4) is essential for mucosal Treg suppressive function and stability. TCR-dependent signals activate mTOR which in turn promotes the expression of IRF4, GATA3 as well as upstream regulators of glycolysis pathway like HK2, Myc, and Foxo (56). Mitochondrial metabolism is highly induced in an mTOR dependent manner during Treg activation since Treg specific depletion of mitochondrial transcription factor resulted in the hyperactivation of Tconv and autoimmunity in mice (56).

It is well-accepted that glycolysis promotes Treg cell growth and migration at the cost of immune suppressive-function. Upon TCR ligation and distinct co-stimulations, transcription factor c-Myc and the hypoxia factor HIF-1α initiate the upregulation of genes encoding molecules important in the glycolysis pathway, whereas Bcl-6 directly downregulates glycolysis and associated pathways (57). Under hypoxic situations, HIF-1α prevent glucose-derived pyruvate from the mitochondrial oxidation, resulting in a gglycolytic shift, which leads to the inhibition of Treg function (43). Human Treg that lose FOXP3 expression upon *in vitro* stimulation preferably differentiated into Th2-like Treg (58), and the direct role of FOXP3 in suppressing type 2 cytokine production in Treg during Treg dysfunction has been confirmed by using the IPEX mutation M370I, a naturally occurring FOXP3 mutant derived from IPEX patients (59). The similar phenomenon was also observed in mice. Bcl6−/− Treg could produce Tconv-like levels of Th2 cytokines and are incapable of controlling Th2-type inflammation (60). The metabolic status of Th2-like Treg is still unclear. PTEN-mediated suppression of PI3K activity is critical for maintaining Treg suppressive function (44). PTEN-deficient Treg have increased glycolytic-rates and a significant reduction of FOXP3 expression. In Treg, restriction of mTORC1 signaling and glycolysis by Ndfip1, a coactivator of Nedd4-family E3 ubiquitin ligases, supports their suppressive function (45). Restriction of mTOR-pathway signaling also increases expression of transcription factor Bcl6 in Treg, which supports Treg stability and suppresses glycolysis potentiated by c-Myc and HIF-1α (57). Stimulation of the CTLA-4 and PD-1 pathways increases FOXP3 expression, stimulates OXPHOS and suppresses the glycolytic-flux in Treg (46). Curiously, glycolysis also supports FOXP3 expression under certain conditions. The glycolytic enzyme Enolase-1 has been shown to be multifunctional. Under glycolytic circumstances, Enolase-1 is forced to the cytoplasm instead of binding FOXP3 in the nucleus, preventing it from suppressing FOXP3 expression in human Treg (47). Glycolysis also favors the activity of transcription repressor enhancer of zeste homolog 2 (EZH2), which promotes FOXP3 expression and is critical for the maintenance of Treg (48). We have shown that treatment of human Treg with a TNF receptor 2 agonist enhanced EZH2 expression, as well as lineage stability (49).

TGF-β functions in a complementary role to FOXP3 in promoting mitochondrial oxidative metabolism and inhibiting glycolysis. FOXP3 expression and OXPHOS activity are closely linked to human Treg intracellular ROS levels. ROS promotes FOXP3 stability in human Treg by increasing the activity of the transcription factor nuclear factor of activated T-cells (NFAT), which binds the CNS2 enhancer of FOXP3 (13, 50, 51). FOXP3 controls downregulation of glycolysis and transcription promoting factor Myc, induction of OXPHOS and increases the electron transfer NAD^+^/NADH ratio. FOXP3 regulates T cell metabolism by suppressing Myc and glycolysis, enhancing OXPHOS and increasing nicotinamide adenine dinucleotide oxidation (52). Additionally, the AMPK-pathway inhibits mTOR signaling and thereby promotes OXPHOS (22). These adaptations allow Treg a metabolic advantage in low-glucose, lactate-rich environments, like in inflammation, as they resist lactate-mediated suppression of T cell function and proliferation. OXPHOS regulators are required for optimal Treg function. Deletion of OXPHOS key-regulators Pgc1α or Sirt3 abrogates Treg-dependent suppressive function. Myocyte enhancer factor 2 (Mef2) activity induces the expression of OXPHOS genes. Interestingly, inhibition of Mef2 with histone deacetylase 9 disproportionally affects Treg compared to Tcon, and deletion increases Treg suppressive function (13). As previously described, HIF-1α binds FOXP3 and thereby reduces suppressive function. Additionally, HIF-1α acts as a switch to promote Treg migration via promotion of glycolysis, at the cost of OXPHOS-driven immunosuppression (43). HIF-1α directs glucose to glycolysis and away from the mitochondria by blocking PDH, leaving mitochondrial metabolism dependent on fatty acids. Especially in hypoxic conditions, this diminishes suppressive function. Thus, OXPHOS regulators are required for optimal Treg function (13).

Treg also express high levels of AMPK, which promotes fatty acid oxidation and inhibits the mTOR mediated glycolysis (22). Stimulation of Treg effector molecules CTLA-4 and PD-1 and expression of FOXP3 suppresses glycolysis and promotes fatty acid oxidation *in vitro* (11). PD-1 actively promotes fatty acid oxidation by upregulation of fatty acid transporter CPT1. CTLA-4 and PD-1 activate PTEN to antagonize PI3K-signaling (6). DEP domain-containing mTOR-interacting protein (DEPTOR), a negative regulator of mTOR, has similar effects as partial inhibition of mTORC1 activity, shifting Treg metabolism toward OXPHOS while stabilizing FOXP3 expression and thereby securing Treg survival and suppressive function (53, 61).

Similar to fatty acid oxidation, fatty acid synthesis activity is required for Treg suppressive function although Treg are not dependent on fatty acid synthesis (62). The mevalonate pathway (Figure 2) aids the upregulation of the suppressive molecules CTLA-4 and ICOS (Inducible co-stimulator). Inhibition of the mevalonate pathway by disruption of mTOR by statins, genetic depletion of RAPTOR or inhibition of 25-hydroxycholesterol or 3-hydroxy-3-methylglutaryl-CoA reductase (HMGR) potently blocks human Treg suppressive activity, which can be reversed by addition of mevalonate (63, 64). Proper Treg cell function is also regulated by the ectoenzyme CD39 expression on human Treg. CD39 produces AMP (adenosine monophosphate) from ATP or ADP (adenosine diphosphate), which is further converted to extracellular adenosine by ectoenzyme CD73. Adenosine subsequently binds to adenosine 2A receptor (A2A) and facilitate Treg generation and suppressive function through adenosine-mediated immune suppression (65).

FOXP3 expression can also be regulated by post-translational modifications, which is closely linked to alterations in metabolism. Acetylation of FOXP3 prevents its degradation and is dependent on the availability of acetyl-CoA, a product of OXPHOS. However, fatty acid oxidation also antagonizes the stability of human Treg by promoting an increased NAD^+^/NADH ratio, which increases the activity of deacetylase SIRT1 (silent mating type information regulation 2 homolog) (66). Short-chain fatty acids, like butyrate, stabilize Treg by preventing histone deacetylase from suppressing FOXP3 expression (28). IDO-mediated tryptophan metabolisms have inhibitory effects on Th1 and Th17 cells, while administration of the downstream tryptophan metabolite 3-hydroxy anthranilic acid (3-HAA) enhanced the percentage of Treg. IDO-deficient mice have reduced Treg levels (33), while IDO expression in plasmacytoid dendritic cells can induce tryptophan degradation and thereby support Treg generation and suppressive function.

In summary, glycolysis is associated with decreased Treg suppressive function. Lipid metabolism favorites Treg lineage stability. Several metabolites including purine, tryptophan, retinoic acid, and glutamine are crucial to support the induction of FOXP3 gene as well as its sustained stable expression (Figure 3C). The high expression of suppressive molecules on Treg like PD-1, CTLA-4, CD39, and AHR are crucial for Treg to sense the nutrient and energy change of its local milieu.

## Treg Metabolism in Pathological Conditions

Alterations in Treg numbers and function have been widely demonstrated in human autoimmune, infectious and allergic diseases and cancers (67). Decreased Treg numbers have been reported in patients with diabetes mellitus type II and has been contributed to both high glucose and high-density lipoprotein concentrations in blood (68). Circulating and visceral adipose Treg are reduced in obese individuals, inversely correlating with measures of adiposity, inflammation and glucose tolerance, enabling identification of subjects at increased metabolic and cardiovascular risk (69). PPARγ signaling for energy homeostasis in Treg maintains adipose-tissue inflammatory tone and insulin sensitivity in lean adipose tissue, while disfunction attenuates insulin-sensitization (26). Interestingly, frequency and suppressive capacity of Treg remains unaltered following silencing of INSR, although attenuation of acute graft-vs.-host disease and multiple sclerosis has been observed in animal models (35). A role for Treg metabolism has also been described in cancer progression, with increases in circulating and tumor-infiltrating Treg strongly associated with advanced cancer stage and poor prognosis. The hypoxic tumor microenvironment supports Treg function and accumulation. Additionally, IDO-expression in the tumor microenvironment has been reported to support the conversion of conventional CD4+ T cells into Treg (70). IDO-expression associated Treg development has also been described in autoimmunity. IDO-deficient mice develop exacerbated experimental autoimmune encephalomyelitis (33) and IDO, together with ARG1, is lower expressed in blood cells of multiple sclerosis patients compared with healthy subjects (71). Increased IDO-expression is generally accompanied by increased mTOR expression, resulting in decreased Treg numbers and increased disease activity. mTOR-controlled pathways are likely to shape autoimmune responses in rheumatic diseases as well (72). In patients with systemic lupus erythematosus, mTORC1 is activated, and mTORC2 is inhibited, with activation of mTORC1 preceding disease flares, and being reduced during successful therapeutic intervention (72). Additionally, an important candidate gene for systemic lupus erythematosus susceptibility has been identified as a major regulator of mitochondrial metabolism and has been shown to reduce Foxp3 expression in Treg (73). Further research to Treg metabolism in pathological conditions, especially using metabolomic-approaches comparing various patient groups and healthy subjects, is imperative to aid a better understanding of the link between functional alterations of Treg and their intracellular metabolism.

## Exploiting Treg Metabolism for Tolerance-Inducing Therapies

A better understanding of Treg metabolism and its distinction from other T cell subsets metabolism allows for the specific modulation of Treg *in vivo* or the improvement of adoptive Treg transfusion therapies. The encompassing goal of such therapies has been the induction of functional immunotolerance by harboring the natural specific immunosuppressive mechanisms without requiring damaging immunosuppressive drugs (74). Treg therapies could improve the current standard of care in the reduction of cost, increased availability, specificity to destructive immune responses, and applicability to different organs. However, before employing such therapies, it is imperative to understand the molecular mechanisms underlying critical Treg functionalities and to identify any factors that confound outcomes. For successful Treg therapy, it is rudimental to acquire a sufficient number of Treg, that these Treg migrate to their desired location and to subsequently have stable immunosuppressive functionality of Treg (75). Treg metabolism could be employed for this (Figure 3). Drugs for modulating cellular metabolism are already available, providing the field of immunometabolism with great opportunity to translate their findings to the clinic (Table 2).

**Table 2:** Metabolic modulators and their relevance for Treg proliferation, migration, and suppressive function.

**Modulator**	**Pathway**	**Effect**
2-DG	Inhibition of glycolysis	Decreases Treg numbers (14, 15, 19, 35) Decreases Treg migration (14, 19, 35)
D-mannose	Inhibition of glycolysis	Increases Treg numbers (76)
DCA	Inhibition of glycolysis	Increases Treg numbers (77)
DASA-58^*^ and TEPP-46^*^	Inhibition of HIF-1α	Increases Treg numbers (78) Increases Treg suppressive function (78)
Mycophenolic acid	Inhibition of guanine nucleotide synthesis	Increases PD-1, CTLA-4, and FOXP3 expression of CD4+ T cells (79)
Dimethyl fumarate	TCA cycle	Increases Treg numbers (80)
UK5099^*^	Inhibition of TCA cycle	Decreases Treg numbers (19)
Rotenone	Inhibition of OXPHOS	Decreases Treg numbers (19) Decreases Treg suppressive function (13)
Oligomycin^*^	Inhibition of OXPHOS	Decreases Treg numbers (19) Decreases Treg suppressive function (22)
Metformin	Increase of FAO, inhibition of OXPHOS	Increases Treg numbers (23) Decreases Treg suppressive function (22)
AICAR	Increase of FAO	Increases Treg numbers (81)
Celastrol	Increase of FAO	Increases Treg numbers (82)
Etomoxir	Inhibition of FAO	Decreases Treg numbers (23)
C75^*^	Inhibition of FAS	Decreases Treg suppressive function (63)
Cerulenin^*^	Inhibition of FAS	Decreases Treg suppressive function (63)
Simvastatin	Inhibition of cholesterol synthesis	Decreases Treg numbers (63)
Amitriptyline	ASM	Increases Treg numbers (83)
BPTES^*^	Inhibition of glutaminolysis	Increases Treg numbers (31)
Rapamycin	Various	Increases Treg numbers (84) Redirects Teff migration (85) Increases Treg suppressive function (84)

*2-DG, 2-deoxy-D-glucose; DCA, dichloroacetate; AICAR, 5-aminoimidazole-4-carboxamide ribonucleotide; BPTES, bis-2-(5-phenylacetamido-1,2,4-thiadiazol-2-yl)ethyl sulfide 3; HIF-1α, hypoxia-inducible factor 1-alpha; TCA, tricarboxylic acid cycle; OXPHOS, oxidative phosphorylation; FAO, fatty acid oxidation; FAS, fatty acid synthesis; ASM, acid sphingomyelinase*.

* *No experimental data specific for modulator available*.

Glycolysis is important for Treg migration. Although most research points toward a negative role for glycolysis in Treg proliferation, it has become clear that complete depletion of glucose from cell culture medium is detrimental for *in vitro* Treg proliferation and suppressive function. Inhibition of glycolysis reduces intracellular pyruvate levels, thereby preventing the conversion to acetyl-CoA via PDHK for mitochondrial oxidative metabolism (Figure 2) and can consequently reduce Treg proliferation (77). Interestingly, glycolysis can potentially be modulated in a Treg-specific manner, as Treg convert glucose to glucose-6-phosphate with a distinct isoform of hexokinase, hexokinase 1 (HK1) (19). At present, pharmacological interference with glycolysis can be obtained through 2-deoxy-D-glucose (2-DG), a glucose analog which is currently used at high concentrations in cancer therapy. 2-DG reportedly inhibits both Treg migration and proliferation (14, 15, 19, 35). Contrastingly, D-mannose, a C-2 epimer of glucose which also inhibits glycolysis, has been described to increase human Treg proliferation *in vitro* by the promotion of TGF-β activity which in turn leads to the increase of fatty acid oxidation (76). Mycophenolic acid, the active ingredient of the immunosuppressant mycophenolate mofetil currently in use for suppressing solid organ rejection, inhibits monophosphate dehydrogenase, an enzyme involved in the biosynthesis of guanine nucleotides, which follows the glycolysis-parallel pathway PPP (79). Interestingly, mycophenolic acid-enhanced expression of PD-1, CTLA-4, and FOXP3 and reduced Akt-mTOR and STAT5 signaling in human CD4^+^ T cells.

Fatty acid oxidation is increased in proliferative Treg and inhibition thereof results in decreased Treg numbers (81). Moreover, oxidative metabolism is associated with and can be induced by Treg suppressive molecules such as TGF-β, CTLA-4, PD-1, and FOXP3. Dimethyl fumarate, a derivate of the TCA cycle intermediate fumarate, stimulates proliferation and development of Treg by supporting mitochondrial oxidative metabolism and FOXP3. Of note, dimethyl fumarate causes lymphopenia and selectively depletes highly glycolytic Teff while sparing oxidative naïve T cells and Treg (80). OXPHOS can be increased by the immunomodulatory metabolite rapamycin, which is used *in vitro* to expand Treg, potently suppresses T cell proliferation and increases Treg suppressive function *in vitro* and *in vivo*. This suggests that it is a valuable drug for adjuvant therapy to improve the efficacy of T(reg)-based immunosuppressive protocols (84). However, rapamycin also redirects Teff peripheral tissue trafficking and stimulates homing to lymph nodes by inhibition of mTORC1 (85). Whether rapamycin also redirects Treg migration is not yet established.

In a situation where blocking Treg function is preferred such as in the tumor milieu, the widely used anti-diabetic drug metformin can be employed. Metformin inhibits the electron transport chain and decreases mitochondrial ROS production, both AMPK-dependent and independent. Metformin increases Treg differentiation, most likely via suppressed activation of mTOR and HIF-1α and stimulation of AMPK and FOXP3 expression (23). Conversely, CPT1 inhibitor etomoxir inhibits fatty acid oxidation, which specifically reduced Treg differentiation and proliferation, although this might be caused by the off-target effects of etomoxir on metabolism. The chines herbal compound celastrol also has immunosuppressive capacities by promoting fatty acid oxidation via upregulation of CPT1 and AMPK expression. Additionally, celastrol has been indicated to facilitate FOXP3 expression and Treg cell generation (82). Pharmacological inhibition of acid sphingomyelinase (ASM), with a clinically used tricyclic antidepressant like amitriptyline, induces higher frequencies of Treg among T cells. This is due to ASM inhibition increasing cell death of T cells in general, while CD25^high^ Treg are protected via IL-2 (83). Further, ASM deficient pTreg have less Akt activity and RICTOR levels compared with control pTreg. Inhibitors of the rate-limiting enzyme HMGR impairs Treg proliferation and function whereas addition of mevalonate, the metabolite downstream of HMGR restores Treg-mediated suppression (63), suggesting that manipulation of lipid biosynthesis, in particular via the mevalonate pathway, would result in Treg functional disruption.

Various studies have described the alterations of metabolic pathways and key metabolic byproducts in several autoimmune disorders. Disease-specific metabolic changes in overall glycolytic activity and oxidative state have been reported in rheumatoid arthritis and multiple sclerosis. In multiple sclerosis, impaired proliferation is suggested to be a consequence of increased levels of circulating leptin. The glutaminolysis pathway has been suggested as a biomarker for disease severity (86). In solid organ transplantation, it is reported that the metabolic environment might influence immune responses and overall transplantation outcome. Lee et al. have shown that by simultaneously blocking glycolysis and the glutamine pathway in the inflammatory transplantation microenvironment, allo-specific Teff responses could safely be reduced while preserving immunoregulation (87). Indeed, both glycolysis and glutamine are associated with a pro-inflammatory phenotype and non-essential for Treg suppressive function, although not irrelevant for Treg proliferation. Wawman and colleagues have described the importance of the hepatic microenvironment in transplantation. The continuous exposure of metabolites and nutrients influences lineage fitness, function, proliferation, migration, and survival of Treg (88). This paves the way for safe and specific novel approaches to modulate the inflammatory environment, for example with tissue-specific accumulation of nanobiologicals (89).

## Concluding Remarks

Knowledge on the role of metabolism in immune cells is rapidly expanding and has increasingly been acknowledged as a potential target for therapies aimed at modulating the immune system either to enhance or suppress immunological responses. Metabolic profiles in Treg are distinctly different between proliferation, migration, and suppressive function, and can be modulated using readily available metabolic and immunomodulatory drugs. However, as metabolic modulation impacts Treg differently throughout their functional profiles, we lack precise insight into the regulatory switches. While modulation of some aspects of glycolysis keeps Treg proliferation and suppressive function intact, an overall increase in glycolysis inhibits Treg suppressive capacity, however, this supports Treg migration. Contrary, oxidative metabolism is crucial to support Treg suppressive function but appears less relevant for migratory behavior. Taken together, this demonstrates the counter-regulation of Treg cell metabolism by pro- and anti-inflammatory signals.

Current technologies for investigating immunometabolism of Treg are limited. Glycolysis and OXPHOS can be measured using Seahorse technology or fluorescent uptake of metabolites, but for the application of these techniques, a high number of cells are required. In the case of Treg, comprising only 1–5% of circulating CD4+ cells, it means that *ex vivo* expansion is almost unavoidable, which may change their metabolic profile. Studying the metabolism of tissue resident-Treg is challenging for similar reasons, with the added challenge purification from tissues brings. To enable research on the metabolism of migrating Treg, improved *in vivo* Treg tracking techniques are required, just as advanced *in vitro* techniques that can combine the technology of migration chambers and metabolism assessment. Also, it should be appreciated that the difference in metabolic profiles, in general, is context-dependent. Distinct Treg proliferative behavior has been reported between *in vitro* and *in vivo* experiments, which indicates the milieu of the microenvironment to be of crucial importance for linking metabolism to immune cell function.

Overall, although exploitation of Treg metabolism seems promising and results could be translated into practice relatively easily, significant challenges are still to be faced. Future research using novel -omics approaches will offer further insight into the molecular mechanisms underlying Treg metabolism. These insights will guide new research on the improvement of current Treg therapies. Given the reliance of specific T cell subsets on certain metabolic pathways, it is possible that the ability of subsets to use and regulate specific pathways is differently regulated and differently metabolic sensitive. Although a simplified reductionistic approach to immunometabolism makes the concept more comprehensible, it is imperative to keep in mind that metabolism is complex, and pathways intertwine and impact each other at many different levels. From a pragmatic point of view, immunometabolism presents excellent possibilities for modulating immune responses, as drugs altering the metabolic state of cells are readily available.

Collectively, the data described in this literature review emphasizes the link between immune cell metabolism and Treg profiles, and underline the importance of understanding the machinery providing the energy required for immune cell functions and could have implications in natural mechanisms to increase Treg suppressive function, like in the transplant setting and autoimmunity. The fields of immunometabolism and Treg research are both burgeoning and combining them might prove useful for patients' benefit in the near future.

## Author Contributions

RK, IJ, HK, and XH contributed conception, design of the study, and wrote sections of the manuscript. RK organized the database and wrote the first draft of the manuscript. All authors contributed to manuscript revision, read, and approved the submitted version.

### Conflict of Interest

The authors declare that the research was conducted in the absence of any commercial or financial relationships that could be construed as a potential conflict of interest.

## Abbreviations

2-DG: 2-deoxy-D-glucose
3-HAA: 3-hydroxy anthranilic acid
A2A: Adenosine 2A receptor
Acetyl-CoA: Acetyl coenzyme A
ADP: Adenosine diphosphate
AHR: Aryl hydrocarbon receptor
AMP: Adenosine monophosphate
AMPK: AMP-activated protein kinase
ARG1: Arginase 1
ASM: Acid sphingomyelinase
ATP: Adenosine triphosphate
CCR7: C-C chemokine receptor 7
CD62L: Leukocyte adhesion molecule L-selectin
CPT1: Carnitine palmitoyltransferase 1
CPT2: Carnitine palmitoyltransferase 2
CTLA-4: Cytotoxic T-lymphocyte-associated protein 4
DEPTOR: DEP domain-containing mTOR-interacting protein
EZH2: Enhancer of zeste homolog 2
FAO: Fatty acid oxidation
Foxo: Forkhead box protein O
FOXP3: Forkhead box p3
GCK: Glucokinase
HIF-1α: Hypoxia-inducible factor 1α
HK: Hexokinase
HMGR: HMG-CoA reductase
ICOS: Inducible co-stimulator
IDO: Indoleamine-pyrrole 2,3-dioxygenase
INSR: Insulin receptor
IRF4: Interferon regulatory factor 4
LDH: Lactate dehydrogenase
LFA-1: Lymphocyte function-association 1
MAPK: Mitogen-activated protein kinase
Mef2: Myocyte enhancer factor 2
mTOR: Mammalian target of rapamycin
mTORC1: Mammalian target of rapamycin complex 1
mTORC2: Mammalian target of rapamycin complex 2
NAD^+^: Oxidized nicotinamide adenine dinucleotide
NADH: Reduced nicotinamide adenine dinucleotide
NADP^+^: Oxidized nicotinamide adenine dinucleotide phosphate
NADPH: Reduced nicotinamide adenine dinucleotide phosphate
Ndfip1: Nedd4-family E3 ubiquitin ligases
NFAT: Nuclear factor of activated T-cells
OXPHOS: Oxidative phosphorylation
PD-1: Programmed cell death protein 1
PDH: Pyruvate dehydrogenase
PDHK1: Pyruvate dehydrogenase kinase 1
PDK1: Phosphoinositide-dependent kinase 1
PI3K: Phosphatidylinositol 3-kinase
PPARγ: Peroxisome proliferator-activated receptors γ
PPP: Pentose phosphate pathway
PTEN: Phosphatase and tensin homolog
pTreg: Periphery-inducible regulatory T cell
RAPTOR: Regulatory-associated protein of mTOR
RICTOR: Rapamycin-insensitive companion of mTOR
ROS: Reactive oxygen species
S1PR1: Sphingosine-1-phosphate receptor 1
SIRT1: Silent mating type information regulation 2 homolog
TCA cycle, Tricarboxylic acid cycle, or citric acid cycle: or Krebs cycle
TCR: T cell receptor
Teff: Effector T cell
TGF-β: Transforming growth factor beta
Treg: Regulatory T cell
tTreg: Thymic-derived regulatory T cell
VAT: visceral adipose tissue.

## References

[1] Fishman JA. Infection in organ transplantation. Am J Transpl. (2017) 17:856–79. 10.1111/ajt.14208 28117944

[2] Net JB Bushell A Wood KJ Harden PN. Regulatory T cells: first steps of clinical application in solid organ transplantation. Transpl Int. (2015) 29:3–11. 10.1111/tri.12608 25981203

[3] Schmidt A Oberle N Krammer PH. Molecular mechanisms of treg-mediated T cell suppression. Front Immunol. (2012) 3:51. 10.3389/fimmu.2012.00051 22566933 PMC3341960

[4] He X Koenen HJPM Slaats JHR Joosten I. Stabilizing human regulatory T cells for tolerance inducing immunotherapy. Immunotherapy. (2017) 9:735–51. 10.2217/imt-2017-0017 28771099

[5] Huynh A DuPage M Priyadharshini B Sage PT Quiros J Borges CM. Control of PI(3) kinase in Treg cells maintains homeostasis and lineage stability. Nat Immunol. (2015) 16:188–96. 10.1038/ni.3077 25559257 PMC4297515

[6] Patsoukis N Bardhan K Chatterjee P Sari D Liu B Bell LN. PD-1 alters T-cell metabolic reprogramming by inhibiting glycolysis and promoting lipolysis and fatty acid oxidation. Nat Commun. (2015) 6:6692. 10.1038/ncomms7692 25809635 PMC4389235

[7] Kawai K Uchiyama M Hester J Wood K Issa F. Regulatory T cells for tolerance. Hum Immunol. (2018) 79:294–303. 10.1016/j.humimm.2017.12.013 29288698

[8] O'Neill LA Kishton RJ Rathmell J. A guide to immunometabolism for immunologists. Nat Rev Immunol. (2016) 16:553–65. 10.1038/nri.2016.70 27396447 PMC5001910

[9] Van der Heiden MG Cantley LC Thompson CB Understanding the Warburg effect: the metabolic requirements of cell proliferation. Science. (2009) 324:1029–33. 10.1126/science.1160809 19460998 PMC2849637

[10] Chang CH Chang CH Curtis JD Maggi LB Faubert B Villarino AV. Posttranscriptional control of T cell effector function by aerobic glycolysis. Cell. (2013) 153:1239–51. 10.1016/j.cell.2013.05.016 23746840 PMC3804311

[11] Gerriets VA Kishton RJ Johnson MO Cohen S Siska PJ Nichols AG. FOXP3 and Toll-like receptor signaling balance Treg cell anabolic metabolism for suppression. Nat. Immunol. (2016) 17:1459–66. 10.1038/ni.3577 27695003 PMC5215903

[12] Koenen HJPM Smeets RL Vink PM Van Rijssen E Boots AMH Joosten I. Human CD25highFoxp3pos regulatory T cells differentiate into IL-17 producing cells. Blood. (2008) 112:2340–52. 10.1182/blood-2008-01-133967 18617638

[13] Beier UH Angelin A Akimova T Wang L Liu Y Xiao H. Essential role of mitochondrial energy metabolism in FOXP3+ T-regulatory cell function and allograft survival. FASEB J. (2015) 29:2315–26. 10.1096/fj.14-268409 25681462 PMC4447222

[14] Kishore M Cheung KCP Fu H Bonacina F Wang G Coe D. Marelli-Berg. Regulatory T cell migration is dependent on glucokinase-mediated glycolysis. Immunity. (2017) 47:875–89.e10. 10.1016/j.immuni.2017.10.017 29166588 PMC5714502

[15] Shi LZ Wang R Huang G Vogel P Neale G Green DR. HIF1α-dependent glycolytic pathway orchestrates a metabolic checkpoint for the differentiation of TH17 and Treg cells. J Exp Med. (2011) 208:1367–76. 10.1084/jem.20110278 21708926 PMC3135370

[16] Clambey ET McNamee EN Westrich JA Glover LE Campbell EL Jedlicka P. Hypoxia-inducible factor-1 alpha-dependent induction of FOXP3 drives regulatory T-cell abundance and function during inflammatory hypoxia of the mucosa. Proc Natl Acad Sci USA. (2012) 109:2784–93. 10.1073/pnas.1202366109 22988108 PMC3478644

[17] Kim JW Tchernyshyov I Semenza GL Dang CV. HIF-1-mediated expression of pyruvate dehydrogenase kinase: a metabolic switch required for cellular adaptation to hypoxia. Cell Metab. (2006) 3:177–85. 10.1016/j.cmet.2006.02.002 16517405

[18] Papandreou I Cairns RA Fontana L Lim AL Denko NC. HIF-1 mediates adaptation to hypoxia by actively downregulating mitochondrial oxygen consumption. Cell Metab. (2006) 3:187–97. 10.1016/j.cmet.2006.01.012 16517406

[19] Gerriets VA Kishton RJ Nichols AG MacIntyre AN Inoue M Ilkayeva O. Metabolic programming and PDHK1 control CD4+ T cell subsets and inflammation. J Clin Invest. (2015) 125:194–207. 10.1172/JCI76012 25437876 PMC4382238

[20] Tarasenko TN Gomez-Rodriguez J Sudderth J DeBerardinis RJ McGuire PJ Pyruvate dehydrogenase deficiency reveals metabolic flexibility in T-cells. Mol Genet Metab. (2018) 123:81–2. 10.1016/j.ymgme.2017.12.430

[21] Procaccini C De Rosa V Galgani M Abanni L Cali G Porcellini A. An oscillatory switch in mTOR kinase activity sets regulatory T cell responsiveness. Immunity. (2010) 33:929–41. 10.1016/j.immuni.2010.11.024 21145759 PMC3133602

[22] Michalek RD Gerriets VA Jacobs SR Macintyre AN MacIver NJ Mason EF. Cutting edge: distinct glycolytic and lipid oxidative metabolic programs are essential for effector and regulatory CD4+ T cell subsets. J Immunol. (2011) 186:3299–303. 10.4049/jimmunol.1003613 21317389 PMC3198034

[23] Lee SY Lee SH Yang EJ Kim EK Kim JK Shin DY. Metformin ameliorates inflammatory bowel disease by suppression of the STAT3 signaling pathway and regulation of the between Th17/Treg balance. PLoS ONE. (2015) 10:e0135858. 10.1371/journal.pone.0135858 26360050 PMC4567351

[24] De Rosa V Procaccini C Cali G Pirozzi G Fontana S Zappacosta S. A key role of leptin in the control of regulatory T cell proliferation. Immunity. (2007) 26:241–55. 10.1016/j.immuni.2007.01.011 17307705

[25] Procaccini C Carbone F Di Silvestre D Brambilla F De Rosa V Galgani M The proteomic landscape of human *ex vivo* regulatory and conventional T cells reveals specific metabolic requirements. Immunity. (2016) 44:406–21. 10.1016/j.immuni.2016.01.028 26885861 PMC4760097

[26] Cipolletta D Feuerer M Li A Kamei N Lee J Shoelson SE. PPAR-gamma is a major driver of the accumulation and phenotype of adipose tissue Treg cells. Nature. (2012) 486:549–53. 10.1038/nature11132 22722857 PMC3387339

[27] Feuerer M Herrero L Cipolletta D Naaz A Wong J Nayer A Lean, but not obese, fat is enriched for a unique population of regulatory T cells that affect metabolic parameters. Nat Med. (2009) 15:930–9. 10.1038/nm.2002 19633656 PMC3115752

[28] Furusawa Y Obata Y Fukuda S Endo TA Nakato G Takahashi D. Commensal microbe-derived butyrate induces the differentiation of colonic regulatory T cells. Nature. (2013) 504:446–50. 10.1038/nature12721 24226770

[29] Singh N Gurav A Sivaprakasam S Brady E Padia R Shi H. Activation of Gpr109a, receptor for niacin and the commensal metabolite butyrate, suppresses colonic inflammation and carcinogenesis. Immunity. (2014) 40:128–39. 10.1016/j.immuni.2013.12.007 24412617 PMC4305274

[30] Arpaia N Campbell C Fan X Dikiy S van der Veeken J de Roos P. Metabolites produced by commensal bacteria promote peripheral regulatory T-cell generation. Nature. (2013) 504:451–5. 10.1038/nature12726 24226773 PMC3869884

[31] Klysz D Tai X Robert PA Craveiro M Cretenet G Oburoglu L. Glutamine-dependent alpha-ketoglutarate production regulates the balance between T helper 1 cell and regulatory T cell generation. Sci Signal. (2015) 8:ra97. 10.1126/scisignal.aab2610 26420908

[32] Cobbold SP Adams E Farquhar CA Nolan KF Howie D Lui KO. Infectious tolerance via the consumption of essential amino acids and mTOR signaling. Proc Natl Acad Sci USA. (2009) 106:12055–60. 10.1073/pnas.0903919106 19567830 PMC2704109

[33] Yan Y Zhang G-X Gran B Fallarino F Yu S Li M. IDO upregulates regulatory T cells via tryptophan catabolite and suppresses encephalitogenic T cell responses in experimental autoimmune encephalomyelitis. J Immunol. (2010) 185:5953–61. 10.4049/jimmunol.1001628 20944000 PMC2998795

[34] Chow Z Banerjee A Hickey MJ. Controlling the fire — tissue-specific mechanisms of effector regulatory T-cell homing. Immunol Cell Biol. (2015) 93:355–63. 10.1038/icb.2014.117 25582339

[35] Fischer HJ Sie C Schumann E Witte AK Dressel R Van Den Brandt J. The insulin receptor plays a critical role in t cell function and adaptive immunity. J Immunol. (2017) 198:1910–20. 10.4049/jimmunol.1601011 28115529

[36] Pompura SL Dominguez-Villar M The PI3K/AKT signaling pathway in regulatory T-cell development, stability, and function. J Leukoc. Biol. (2018) 103:1065–76. 10.1002/jlb.2mir0817-349r 29357116

[37] Finlay D Cantrell D. Phosphoinositide 3-kinase and the mammalian target of rapamycin pathways control T cell migration. Ann N Y Acad Sci. (2010) 1183:149–57. 10.1111/j.1749-6632.2009.05134.x 20146713 PMC3520021

[38] Carlson CM Endrizzi BT Wu J Ding X Weinreich MA Walsh ER. Kruppel-like factor 2 regulates thymocyte and T-cell migration. Nature. (2006) 442:299–302. 10.1038/nature04882 16855590

[39] Chen LC Nicholson YT Rosborough BR Thomson AW Raimondi G. A novel mTORC1-dependent, Akt-independent pathway differentiates the gut tropism of regulatory and conventional CD4 T cells. J Immunol. (2016) 197:1137–47. 10.4049/jimmunol.1600696 27402696 PMC4975979

[40] Liu G Burns S Huang G Boyd K Proia RL Flavell RA. The receptor S1P1 overrides regulatory T cell-mediated immune suppression through Akt-mTOR. Nat Immunol. (2009) 10:769–77. 10.1038/ni.1743 19483717 PMC2732340

[41] Priceman SJ Shen S Wang L Deng J Yue C Kujawski M. S1PR1 is crucial for accumulation of regulatory T cells in tumors via STAT3. Cell Rep. (2014) 6:992–9. 10.1016/j.celrep.2014.02.016 24630990 PMC3988983

[42] Opitz CA Litzenburger UM Sahm F Ott M Tritschler I Trump S. An endogenous tumour-promoting ligand of the human aryl hydrocarbon receptor. Nature. (2011) 478, 197–203. 10.1038/nature10491 21976023

[43] Miska J Lee-Chang C Rashidi A Muroski ME Chang AL Lopez-Rosas A. HIF-1α is a metabolic switch between glycolytic-driven migration and oxidative phosphorylation-driven immunosuppression of tregs in glioblastoma. Cell Rep. (2019) 27:226–37.e4. 10.1016/j.celrep.2019.03.029 30943404 PMC6461402

[44] Walsh PT Buckler JL Zhang J Gelman AE Dalton NM Taylor DK. PTEN inhibits IL-2 receptor-mediated expansion of CD4+ CD25+ Tregs. J Clin Invest. (2006) 116:2521–31. 10.1172/jci28057 16917540 PMC1550279

[45] Layman AAK Deng G O'Leary CE Tadros S Thomas RM Dybas JM. NDFIP1 restricts mTORC1 signalling and glycolysis in regulatory T cells to prevent autoinflammatory disease. Nat Commun. (2017) 8:15677. 10.1038/ncomms15677 28580955 PMC5465375

[46] Priyadharshini B Loschi M Newton RH Zhang JW Finn KK Gerriets VA. Cutting edge. TGF-beta and phosphatidylinositol 3-kinase signals modulate distinct metabolism of regulatory T cell subsets. J Immunol. (2018) 201:2215–9. 10.4049/jimmunol.1800311 30209190 PMC6179917

[47] De Rosa V Galgani M Porcellini A Colamatteo A Santopaolo M Zuchegna C. Glycolysis controls the induction of human regulatory T cells by modulating the expression of FOXP3 exon 2 splicing variants. Nat Immunol. (2015) 16:1174–84. 10.1038/ni.3269 26414764 PMC4868085

[48] DuPage M Chopra G Quiros J Rosenthal WL Morar MM Holohan D. The chromatin-modifying enzyme Ezh2 is critical for the maintenance of regulatory T cell identity after activation. Immunity. (2015) 42:227–38. 10.1016/j.immuni.2015.01.007 25680271 PMC4347854

[49] Urbano PCM Koenen HJPM Joosten I He X. An autocrine TNFalpha-tumor necrosis factor receptor 2 loop promotes epigenetic effects inducing human treg stability *in vitro*. Front Immunol. (2018) 9:573. 10.3389/fimmu.2018.00573 29619032 PMC5871762

[50] Li X Liang Y LeBlanc M Benner C Zheng Y. Function of a FOXP3 cis-element in protecting regulatory T cell identity. Cell. (2014) 158:734–48. 10.1016/j.cell.2014.07.030 25126782 PMC4151505

[51] Sena LA Li S Jairaman A Prakriya M Ezponda T Hildeman DA. Mitochondria are required for antigen-specific T cell activation through reactive oxygen species signaling. Immunity. (2013) 38:225–36. 10.1016/j.immuni.2012.10.020 23415911 PMC3582741

[52] Angelin A Gil-de-Gomez L Dahiya S Jiao J Guo L Levine MH. FOXP3 reprograms T cell metabolism to function in low-glucose, high-lactate environments. Cell Metab. (2017) 25:1282. 10.1016/j.cmet.2016.12.018 28416194 PMC5462872

[53] Wedel J Bruneau S Liu K Kong SW Sage PT Sabatini DM. DEPTOR modulates activation responses in CD4+ T cells and enhances immunoregulation following transplantation. Am J Transpl. (2018) 19:77–88. 10.1111/ajt.14995 29969188 PMC6310634

[54] Finlay DK Sinclair LV Feijoo C Waugh CM Hagenbeek TJ Spits H Phosphoinositide-dependent kinase 1 controls migration and malignant transformation but not cell growth and proliferation in PTEN-null lymphocytes. J Exp Med. (2009) 206:2441–54. 10.1084/jem.20090219 19808258 PMC2768858

[55] Haas R Smith J Rocher-Ros V Nadkarni S Montero-Melendez T D'Acquisto F. Lactate regulates metabolic and pro-inflammatory circuits in control of T cell migration and effector functions. PLoS Biol. (2015) 13:e1002202. 10.1371/journal.pbio.1002202 26181372 PMC4504715

[56] Chapman NM Zeng H Nguyen TLM Wang Y Vogel P Dhungana Y. mTOR coordinates transcriptional programs and mitochondrial metabolism of activated Treg subsets to protect tissue homeostasis. Nat Commun. (2018) 9:2095. 10.1038/s41467-018-04392-5 29844370 PMC5974344

[57] Buck MD O'Sullivan D Pearce EL. T cell metabolism drives immunity. J Exp Med. (2015) 212:1345–60. 10.1084/jem.20151159 26261266 PMC4548052

[58] Hansmann L Schmidl C Kett J Steger L Andreesen R Hoffmann P. Dominant Th2 differentiation of human regulatory T cells upon loss of FOXP3 expression. J Immunol. (2012) 188:1275–82. 10.4049/jimmunol.1102288 22210907

[59] Van Gool F Nguyen MLT Mumbach MR Satpathy AT Rosenthal WL Giacometti S. A mutation in the transcription factor Foxp3 drives T helper 2 effector function in regulatory T cells. Immunity. (2019) 50:362–377.e6. 10.1016/j.immuni.2018.12.016 30709738 PMC6476426

[60] Sawant DV Wu H Yao W Sehra S Kaplan MH Dent AL. The transcriptional repressor Bcl6 controls the stability of regulatory T cells by intrinsic and extrinsic pathways. Immunology. (2015) 145:11–23. 10.1111/imm.12393 25262912 PMC4405320

[61] Howie D Cobbold SP Adams E Ten Bokum A Necula AS Zhang W. FOXP3 drives oxidative phosphorylation and protection from lipotoxicity. JCI Insight. (2017) 2:e89160. 10.1172/jci.insight.89160 28194435 PMC5291728

[62] Cluxton D Petrasca A Moran B Fletcher JM. Differential regulation of human Treg and Th17 cells by fatty acid synthesis and glycolysis. Front Immunol. (2019) 10:115. 10.3389/fimmu.2019.00115 30778354 PMC6369198

[63] Zeng H Yang K Cloer C Neale G Vogel P Chi H. mTORC1 couples immune signals and metabolic programming to establish T(reg)-cell function. Nature. (2013) 499:485–90. 10.1038/nature12297 23812589 PMC3759242

[64] Thurnher M Gruenbacher G. T lymphocyte regulation by mevalonate metabolism. Sci Signal. (2015) 8:re4. 10.1126/scisignal.2005970 25829448

[65] Mandapathil M Hilldorfer B Szczepanski MJ Czystowska M Szajnik M Ren J. Generation and accumulation of immunosuppressive adenosine by human CD4+CD25highFOXP3+ regulatory T cells. J Biol Chem. (2010) 285:7176–86. 10.1074/jbc.M109.047423 19858205 PMC2844167

[66] Wellen KE Hatzivassiliou G Sachdeva UM Bui TV Cross JR Thompson CB. ATP-citrate lyase links cellular metabolism to histone acetylation. Science. (2009) 324:1076–80. 10.1126/science.1164097 19461003 PMC2746744

[67] Yang WY Shao Y Lopez-Pastrana J Mai J Wang H Yang X-F. (2015). Pathological conditions re-shape physiological Tregs into pathological Tregs. Burns Trauma. 3:1. 10.1186/s41038-015-0001-0 26623425 PMC4662545

[68] Zhang C Xiao C Wang P Xu W Zhang A Li Q. The alteration of Th1/Th2/Th17/Treg paradigm in patients with type 2 diabetes mellitus. Relationship with diabetic nephropathy. Hum Immunol. (2014) 75:289–96. 10.1016/j.humimm.2014.02.007 24530745

[69] Wagner NM Brandhorst G Czepluch F Lankeit M Eberle C Herzberg S. Circulating regulatory T cells are reduced in obesity and may identify subjects at increased metabolic and cardiovascular risk. Obesity. (2013) 21:461–8. 10.1002/oby.20087 23592653

[70] Wang H Franco F Ho PC. Metabolic regulation of Tregs in cancer. Opportunities for immunotherapy Trends Cancer. (2017) 3:583–92. 10.1016/j.trecan.2017.06.005 28780935

[71] Negrotto L Correale J. Amino acid catabolism in multiple sclerosis affects immune homeostasis. J Immunol. (2017) 198:1900–9. 10.4049/jimmunol.1601139 28130499

[72] Perl A. Activation of mTOR (mechanistic target of rapamycin) in rheumatic diseases. Nat Rev Rheumatol. (2016) 12:169–82. 10.1038/nrrheum.2015.172 26698023 PMC5314913

[73] Choi SC Hutchinson TE Titov AA Seay HR Li S Brusko TM. The lupus susceptibility gene *Pbx1* regulates the balance between follicular helper T cell and regulatory T cell differentiation. J Immunol. (2016) 197:458–69. 10.4049/jimmunol.1502283 27296664 PMC4935607

[74] Issa F Wood KJ. Translating tolerogenic therapies to the clinic - where do we stand? Front Immunol. (2012) 3:254. 10.3389/fimmu.2012.00254 22934094 PMC3422982

[75] Romano M Tung SL Smyth LA Lombardi G. Treg therapy in transplantation: a general overview. Transpl Int. (2017) 30:745–53. 10.1111/tri.12909 28012226

[76] Zhang D Chia C Jiao X Jin W Kasagi S Wu R. D-mannose induces regulatory T cells and suppresses immunopathology. Nat Med. (2017) 23:1036–45. 10.1038/nm.4375 28759052 PMC12180587

[77] Makita N Ishiguro J Suzuki K Nara F. Dichloroacetate induces regulatory T-cell differentiation and suppresses Th17-cell differentiation by pyruvate dehydrogenase kinase-independent mechanism. J Pharm Pharmaco. (2017) 69:43–51. 10.1111/jphp.12655 27757958

[78] Palazon A Goldrath AW Nizet V Johnson RS. HIF transcription factors, inflammation, and immunity. Immunity. (2014) 41:518–28. 10.1016/j.immuni.2014.09.008 25367569 PMC4346319

[79] He X Smeets RL Koenen HJ Vink PM Wagenaars J Boots AM. Mycophenolic acid-mediated suppression of human CD4+ T cells: more than mere guanine nucleotide deprivation. Am J Transpl. (2011) 11:439–49. 10.1111/j.1600-6143.2010.03413.x 21342445

[80] Kornberg MD Bhargava P Calabresi P Snyder SH Dimethyl fumarate mediates immune modulation by inhibition of GAPDH and aerobic glycolysis. Mult Scler. (2017) 23:28–9. 10.1177/1352458517693959

[81] Gualdoni GA Mayer KA Goschl L Boucheron N Ellmeier W Zlabinger GJ. The AMP analog AICAR modulates the Treg/Th17 axis through enhancement of fatty acid oxidation. FASEB J. (2016) 30:3800–9. 10.1096/fj.201600522R 27492924

[82] Zhang J Shan J Chen X Li S Long D Li Y. Celastrol mediates Th17 and Treg cell generation via metabolic signaling. Biochem Biophys Res Commun. (2018) 497:883–9. 10.1016/j.bbrc.2018.02.163 29476742

[83] Schneider-Schaulies J Beyersdorf N. CD4+ FOXP3+ regulatory T cell-mediated immunomodulation by anti-depressants inhibiting acid sphingomyelinase. Biol Chem. (2018) 399:1175–82. 10.1515/hsz-2018-0159 29908119

[84] Hester J Schiopu A Nadig SN Wood KJ. Low-dose rapamycin treatment increases the ability of human regulatory T cells to inhibit transplant arteriosclerosis *in vivo*. Am J Transpl. (2012) 12:2008–16. 10.1111/j.1600-6143.2012.04065.x 22500984 PMC3440570

[85] Sinclair LV Finlay D Feijoo C Cornish GH Gray A Ager A. Phosphatidylinositol-3-OH kinase and nutrient-sensing mTOR pathways control T lymphocyte trafficking. Nat Immunol. (2008) 9:513–21. 10.1038/ni.1603 18391955 PMC2857321

[86] Freitag J Berod L Kamradt T Sparwasser T. Immunometabolism and autoimmunity. Immunol Cell Biol. (2016) 94:925–34. 10.1038/icb.2016.77 27562063

[87] Lee CF Lo YC Cheng CH Furtmuller GJ Oh B Andrade-Oliveira V. Preventing allograft rejection by targeting immune metabolism. Cell Rep. (2015) 13:760–70. 10.1016/j.celrep.2015.09.036 26489460 PMC4626381

[88] Wawman RE Bartlett H Oo YH. Regulatory T cell metabolism in the hepatic microenvironment. Front Immunol. (2018) 8:1889. 10.3389/fimmu.2017.01889 29358934 PMC5766647

[89] Braza MS van Leent MM Lameijer M Sanchez-Gaytan BL Arts RJ Pérez-Medina C. Inhibiting inflammation with myeloid cell-specific nanobiologics promotes organ transplant acceptance. Immunity. (2018) 49:819–28. e6. 10.1016/j.immuni.2018.09.008 30413362 PMC6251711

