# Supplementary material for: Metabolic Pathways Involved in Regulatory T Cell Functionality
Source: Front Immunol. 2019 Dec 3;10:2839. doi: 10.3389/fimmu.2019.02839 (PMC6902900; doi:10.3389/fimmu.2019.02839)
Supplement: Supplementary file 1 [file Table_1.docx]

**Supplementary Table S1.** PubMed and Embase search strategies for articles in English indexed before April 28^th^, 2019 on metabolic pathways involved in Treg functionality.

| PubMed |  |
| --- | --- |
| 1. (Regulatory) T cells  *(35,474 results)* | T-lymphocytes, regulatory [MeSH] OR Immunoregulatory T cell[tiab] OR Immunoregulatory T lymphocyte[tiab] OR Regulatory T cell[tiab] OR Regulatory T lymphocyte[tiab] OR T regulatory cell[tiab] OR T regulatory lymphocyte[tiab] OR Treg[tiab] |
| 2. Immunometabolism  *(2,267,447)* | Metabolism [MeSH] OR Amino acid metabolism[tiab] OR Cell metabolism[tiab] OR Cellular metabolism[tiab] OR Fatty acid metabolism[tiab] OR Fatty acid oxidation[tiab] OR Glucose metabolism[tiab] OR Glutaminase[tiab] OR Glutamine metabolism[tiab] OR Glutaminolysis[tiab] OR Glutathione metabolism[tiab] OR Glycolysis[tiab] OR Krebs cycle[tiab] OR Lipid metabolism[tiab] OR Metabolic reprogramming[tiab] OR Mevalonate metabolism[tiab] OR Oxidative phosphorylation[tiab] OR OXPHOS[tiab] OR Pentose phosphate pathway[tiab] OR TCA cycle[tiab] OR Tricarboxylic acid cycle[tiab] |
| 3. Proliferation  *(544,964 results)* | Cell proliferation [MeSH] OR Cell division [MeSH] OR Cell division[tiab] OR Cellular division[tiab] OR Cell proliferation[tiab] OR Lymphocyte proliferation[tiab] OR Cell expansion[tiab] |
| 4. Migration  *(312,367 results)* | Cell movement [MeSH] OR Leukocyte migration[tiab] OR Lymphocyte migration[tiab] OR Cell migration[tiab] OR Cellular migration[tiab] OR Migration[tiab] |
| 5. Suppressive function  *(327,235 results)* | Immunomodulation [MeSH] OR Suppressive function[tiab] OR Suppressor function[tiab] OR Suppressive capacity[tiab] OR Cytokine production[tiab] OR Suppression assay[tiab] |
| Total | 1 and 2 and (3 or 4 or 5) = 678 results |

| Embase |  |
| --- | --- |
| 1. (Regulatory) T cells  *(60,477 results)* | Exp Regulatory T lymphocyte/ OR Immunoregulatory T cell.mp. OR Immunoregulatory T lymphocyte.mp. OR Regulatory T cell.mp. OR Regulatory T lymphocyte.mp. OR T regulatory cell.mp. OR T regulatory lymphocyte.mp. OR Treg.mp. |
| 2. Immunometabolism  *(289,898 results)* | Exp Cell metabolism/ OR Amino acid metabolism.mp. OR Cell metabolism.mp. OR Cellular metabolism.mp. OR Fatty acid metabolism.mp. OR Fatty acid oxidation.mp. OR Glucose metabolism.mp. OR Glutaminase.mp. OR Glutamine metabolism.mp. OR Glutaminolysis.mp. OR Glutathione metabolism.mp. OR Glycolysis.mp. OR Krebs cycle.mp. OR Lipid metabolism.mp. OR Metabolic reprogramming.mp. OR Mevalonate metabolism.mp. OR Oxidative phosphorylation.mp. OR OXPHOS.mp. OR Pentose phosphate pathway.mp. OR TCA cycle.mp. OR Tricarboxylic acid cycle.mp. |
| 3. Proliferation  *(726,696 results)* | Exp Cell proliferation/ OR exp Lymphocyte proliferation/ OR exp Cell expansion/ OR exp Cell division/ OR Cell division.mp. OR Cellular division.mp. OR Cell proliferation.mp. OR Lymphocyte proliferation.mp. OR Cell expansion.mp. |
| 4. Migration  *(369,352 results)* | Exp migration/ OR Leukocyte migration.mp. OR Lymphocyte migration.mp. OR Cell migration.mp. OR Cellular migration.mp. OR Migration.mp. |
| 5. Suppressive function  *(207,555 results)* | Exp Immunomodulation/ OR Suppressive function.mp. OR Suppressor function.mp. OR Suppressive capacity.mp. OR Cytokine production.mp. OR Suppression assay.mp. |
| Total | 1 and 2 and (3 or 4 or 5) = 406 results |
